# Comparative Genomics of 42 *Arcanobacterium phocae* Strains

**DOI:** 10.3390/antibiotics10060740

**Published:** 2021-06-18

**Authors:** Kirsi J. Aaltonen, Ravi Kant, Nanett Kvist Nikolaisen, Mikkel Lindegaard, Mirja Raunio-Saarnisto, Lars Paulin, Olli Vapalahti, Tarja Sironen

**Affiliations:** 1 Department of Veterinary Biosciences, Faculty of Veterinary Medicine, University of Helsinki, Mustialankatu 1, 00790 Helsinki, Finland; ravi.kant@helsinki.fi (R.K.); olli.vapalahti@helsinki.fi (O.V.); tarja.sironen@helsinki.fi (T.S.); 2 Department of Virology, Faculty of Medicine, University of Helsinki, Mustialankatu 1, 00790 Helsinki, Finland; 3 National Food Institute, Technical University of Denmark, 2800 Kongens Lyngby, Denmark; ml@mixs.dk (M.L.); nannik@food.dtu.dk (N.K.N.); 4 Finnish Food Authority, P.O. Box 100, 00027 Seinäjoki, Finland; mirja.raunio-saarnisto@ruoka.fi; 5 Institute of Biotechnology, University of Helsinki, 00790 Helsinki, Finland; lars.paulin@helsinki.fi; 6 Hospital District of Helsinki and Uusimaa, 00029 Helsinki, Finland

**Keywords:** *Arcanobacterium phocae*, FENP, genomics, emergence

## Abstract

For the last 13 years, the fur industry in Europe has suffered from epidemic spouts of a severe necrotizing pyoderma. It affects all species currently farmed for fur and causes animal welfare problems and significant losses to the farmers. The causative agent of this disease was identified as *Arcanobacterium phocae*. Previously, this bacterium has been isolated from seals and other marine mammals, apparently causing wound and lung infections. Attempts at antibiotic treatment have been unsuccessful and the current advice on preventing the disease is to cull all animals with clinical signs. This poses an urgent question regarding possible vaccine development, as well as the need for further understanding of the pathogenicity of this organism. This study compared the whole genomes of 42 *A. phocae* strains isolated from seals, blue foxes, finnraccoons, mink and otter. The sequences were created using the Illumina technology and annotations were done using the RAST pipeline. A phylogenetic analysis identified a clear separation between the seal strains and the fur-animal-derived isolates, but also indicated that the bacterium readily adapts to new environments and host species with reasonable diversity. A pan- and core-genome was created and analyzed for proteins. A further analysis identified several virulence factors as well as multiple putative and secreted proteins of special interest for vaccine development.

## 1. Introduction

In 2007, a new and emerging disease affecting fur animals was detected in Finland, and then elsewhere in Europe. This disease is a contagious, necrotizing pyoderma, which causes severe animal welfare problems. It affects all fur animal species grown in Finland, but the signs of disease seem to differ according to species. Mink (*Neovison vison*) tend to have necropurulent lesions in their paws but also on facial skin. The fox (*Vulpes lagopus*) suffers from inflammation of the periorbital skin, which then progresses into severe inflammation of the surrounding area. The finnraccoon (*Nyctereutes procyonioides*) develop painful pustules between the toes [1]. The disease has been described, and the causative agent preliminarily identified as *Arcanobacterium phocae*. The etiology of this multifactorial disease-causing necrotizing pyoderma was partially uncovered later through an infection experiment [1,2]. The disease is highly contagious and shows poor response to antibiotic treatment, and the best practice has been to cull the affected animals to stop the epidemic. In vitro, all the tested isolates have been susceptible to antibiotics, but in vivo only prophylactic antibiotics have an effect, and this is forbidden by Finnish law. [1]. To date, the disease and *A. phocae* have been recorded in Finland, Spain, Denmark, Iceland, Norway, the Netherlands, and Canada [3,4,5,6,7]

The earliest mention of similar symptoms in fur animals is from 1996 in Canada, but the causative agent was unidentified at that time. At that point, the onset of symptoms seemed to be connected to feeding the mink with seal meat [3].

*Arcanobacterium phocae* was originally isolated from skin lesions or abscesses in seals and other marine mammals. The first isolates were obtained from grey seals (*Halichoerus grypus*) on the coast of Scotland [8]. The bacteria have since been isolated from marine mammals from the coastal waters of The United States of America as well [9]. They have also been reported from seals, mink and otter from the Danish coast and Spain [10]. The bacterium is a beta-haemolytic, non-motile Gram-positive coccobacillus [9]. Arcanobacteria are commonly found on mucous membranes and traditionally considered to be opportunistic pathogens. However, many well-known pathogens do belong to this species, as well as the Trueperella spp., which was recently separated as a species from Arcanobacteria [11].

The aim of this study was to further characterize the population of *A. phocae* currently circulating in diseased fur animals in Europe and to compare them with isolates from marine mammals. This was a joint effort between the University of Helsinki and the Technical University of Denmark. The analysis was done with 42 whole genomes of *A. phocae*, and the goal was to find factors enabling the known species jump and pathogenicity through whole-genome sequencing, and pan- and core-genome analysis.

## 2. Materials and Methods

### 2.1. Bacterial Isolates, Growth Conditions and DNA Extraction

The isolates included in this study were collected from commercial European fur animals, and Danish wildlife animals. Two methods were used to obtain the sequences. The first method (Method A) was used in 12 isolates from two finnraccoons (*Nyctereutes procyonoides*), two bluefoxes (*Vulpes lagopus*) and eight mink (*Neovison vison*). The second method (Method B) was used on 29 isolates from mink (*n* = 23) originating from various countries (Spain 3, Netherlands 3, Finland 5, Denmark 12), and one grey seal (*Halichoerus grypus*), three spotted seals (*Phoca largha*), one otter (*Lutra lutra*), and one finnraccoon (*Nyctereutes procyonoides*) from Denmark. The type strain [DSM 10,002] originating from grey seal was also included in the analyses [8]. The origin of the strains and their characteristics are listed in Supplementary Table S2. All animals presented with signs of disease, mostly infected skin lesions.

#### 2.1.1. Growth and DNA Extraction Method A

The Finnish fur animal isolates were grown on bloodagar plates with 4% defibrinated sheep blood for 48 h. The bacteria were harvested by scraping the cells from the plates and collecting in microcentrifuge tubes. Epicentre by Lucigen MasterPure Gram Positive DNA Purification Kit (Lucigen Corp., Middleton, WI, USA) was used to extract DNA. The bacteria were stored at -20 °C prior to extraction. Kit instructions were followed, with the optional lysozyme treatment used overnight. The DNA was analyzed with Bioanalyzer (Agilent, Santa Clara, CA, USA) to verify quality and concentration.

#### 2.1.2. Growth and DNA extraction Method B

The bacteria were isolated from clinical material from mink *(Neovison vison)*, finnraccoon (*Nyctereutes* procyonoides), otter (*Lutra lutra*), spotted seal (*Phocae largha*), and grey seal (*Halichoerus grypus*) in the period 2013–2017. All isolates were stored at −80 °C up until DNA extraction. 

The bacteria were grown on Columbia agar plates supplemented with 5% calf blood at 37 °C for up to 48 h. They were harvested by scraping and the DNA was isolated by Maxwell^®^ equipment, using the 16 LEV Blood DNA Kit according to the manufacturer’s instructions (Promega Corporation, Madison, WI, USA) with an additional lysostaphin treatment, as previously performed by [12]. The DNA were analyzed with NanoDrop (NanoDrop Technologies, Wilmington, DE, USA) and Qubit (Life Technologies, Carlsbad, CA, USA) to verify sufficient quality and concentration.

### 2.2. Genome Sequencing and Annotation

#### 2.2.1. Method A

The Illumina platform was used to sequence the 12 *A. phocae* strains. The Bioruptor NGS sonicator (Diagenode, Denville, NJ, USA) was used to shear 0.5 µg of the genomic DNA to 600 bp parts, and these were then analyzed for quality. A-tails were generated and TruSeq adapter was ligated. MPure XP beads (Agencourt, Beckman Coulter) were used to purify the ligation. The library preparation PCRs were performed with Phusion Hot Start II DNA Polymerase (Thermo Fisher). The Index P7 primers were chosen with Barcosel and full-length P5 primers were added. AMPure XP beads were used to purify the reactions. They were pooled and selected for size, as described before by [13]. The Miseq Sequencer and v3600 cycle kit (Illumina, San Diego, CA, USA) were used to sequence the library.

#### 2.2.2. Method B

Sequences created under method B were purchased as a service. They were created at Statens Serum Institut, Denmark. They used the Illumina platform and the NextEra XT sample preparation kit (Illumina, San Diego, CA, USA).

Genomes of the 41 newly sequenced (method A and B) *A. phocae* strains were deposited in GenBank, with the accession numbers listed in Supplementary Table S2. The assembled DNA sequences were used to perform the annotations for the draft genomes. An automatic annotation pipeline Rapid Annotation using Subsystem Technology (RAST) [14] was used to analyze the genomes, and the results were manually curated when needed.

### 2.3. Orthologous Gene Prediction and Genome Sequence Comparison

The identification of orthologous genes for the 42 *A. phocae* genomes was pdone by an all-against-all comparison of the annotated genes of all the genomes using blastp [15]. A standard scoring matrix BLOSUM62 and an *E*-value cut-off of 1 × 10^−5^ were used. 

The blast bit scores were compared against the best possible bit score, scaling the score ratio value (SRV) between 0 and 100. This is a superior indicator of the quality of the hits compared to the raw blast bit score as described by [16].

The orthology of genes was accepted if a reciprocal best blast hit could be shown between them and they showed an SRV of above 32. This threshold value is calculated from the distribution of blast hits between analyzed sequences according to an earlier protocol [17]. Based on this, the core genome was formed to include the set of genes that had orthologies in all the analyzed strains.

The pan-genome was correspondingly calculated to hold all the unique genes in all the analyzed strains. All genes of one chosen reference genome were matched, one by one, with the genes of the rest of the genomes. Any genes that had no orthologies in the starting set of genes were added to the set of pan-genome.

### 2.4. Phylogenetic Construction

A modified version of the pipeline published by [18] was used to construct the phylogenic tree. Alignments of the core gene sets were compiled using MUSCLE [19]. The multiple alignments were trimmed and excluded using the GBLOCKS [20]. A phylogenetic tree was created by the trimmed, multiple-alignment system using the neighbour-joining method of PHYLIP [21].

### 2.5. Identification of the Putative Secreted Proteins as well as the Putative Virulence Factors in the Genomes

Secreted proteins with signal peptides, lipoproteins and the Tat signal peptides transported by the Tat translocon were predicted by the SignalP-5.0 program (https://services.healthtech.dtu.dk/service.php?SignalP (accessed on August 2019)) [22]. The Virulence Factor Database (VFDB) and subcategory of Gram-positive bacteria [23], as well as previously published virulence factors of *A. phocae,* were used to pre-select the factors to look for. The Virulence Factor Database is comprised of scientifically validated or suspected bacterial virulence factors from clinically important bacterial species. Two methods were used to analyze the virulence factors: the core genome and the annotated pangenome. This identified both the most essential virulence factors as well as the more dispensable ones.

## 3. Results and Discussion

### 3.1. General Features of the Genomes of 42 Arcanobacterium phocae Isolates

In this study, we have constructed the genomes of 41 *A. phocae* isolates via high-throughput sequencing and we have additionally obtained the genome of the original isolate from Genbank for comparison. The draft sequences were assembled and initially annotated using an automated pipeline for gene identification, and then manually curated for better quality. Plasmid sequences were excluded from this annotation process. A list of the annotated genes predicted for the 41 newly sequenced genomes is given as supporting information (Supplementary Table S1), with each genomic sequence deposited into GenBank (Supplementary Table S2). The general features of the 42 genomes, as well as the origin of each isolate included and analyzed in this study are presented in Supplementary Table S1. Bacterial genomes were characterized from mink (31), finnraccoon (3), bluefox (2), otter (1), spotted seal (3), and grey seal (1) hosts. 

The 41 genomes generated in this study are draft assemblies, but they represent good-quality sequence data for the performance of genomic comparisons (Figure S1). The type strain sequence obtained from Genbank was a complete genome from a British isolate originating from the lung of a seal [8]. The number of contigs in the genomes assembled in this study varied between 11 and 207 (20,171 and APH96, respectively). The genome size ranged between 1.86 (IN-5A and 20,171) and 2.05 (APH96) Mbps. The total GC content varied only very slightly and remained between 49.9% and 50.6%. The numbers of predicted protein-encoding open reading-frames (ORFs) in the 42 analyzed isolates varied from 1636 (IN-5A (mink) and sample 20,171 (otter)) to 2091 (APH96 (mink)), suggesting reasonable diversity in *A. phocae* as a species. However, the variance between fur animal isolates and seal derived isolates, respectively, was far lower, making isolates from same host species more alike. The strains representing the highest and lowest values in many of these categories seem to be the same strains as the mink IN-5A and otter 20,171, representing lower end of contigs, genome size and ORFs and finnraccoon APH96, representing the upper range in all these evaluations.

### 3.2. Phylogeny

**Figure 1:**
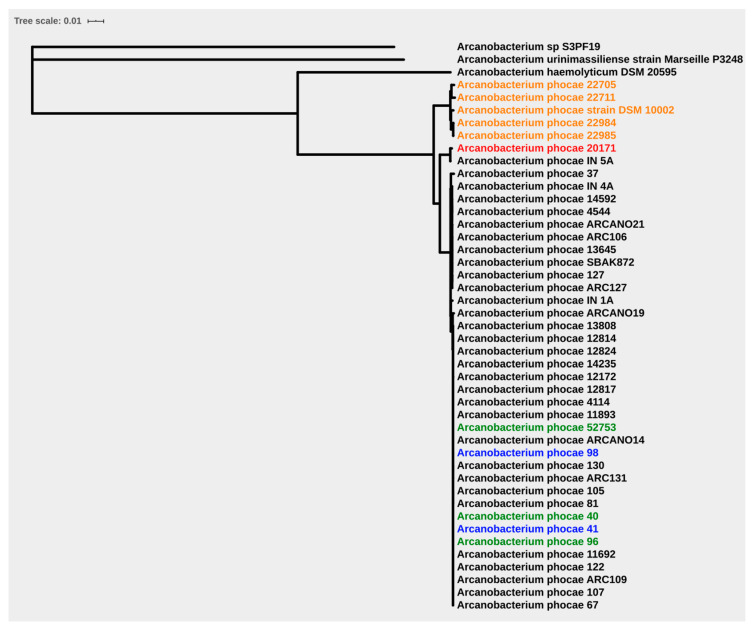
Phylogenetic tree based on core genome (Mink in black color, Finn-raccoon in green, Bluefox in blue, seal in orange and otter in red) (1296 genes).

Constructing a core-genome-based phylogenetic tree from our 42 strains offers an extra understanding of the peripheral phyletic relations and divergent origins by offering possible correlations. A comparison of the genomes illustrated in Supplementary Figure S1 show the strain diversity. A phylogenetic tree of the 42 strains was constructed using the multiple alignment of 1296 core proteins, as illustrated in Figure 1. The strains are all very similar, but we can identify three clusters, with the seal strains (number and name of the seal strains) in one and the fur-animal-derived strains in another. The third cluster is formed of only two strains (IN 5A, 20,171), which fall genetically between the two other clusters. These two are the isolate from an otter from Denmark and a mink from Spain, which, again, are samples IN-5A and 20,171, which also stood out when analyzing the quality and characteristics of the sequences. This suggests that there may have only been a single introduction to the fur animals, possibly during the primary epidemic in Canada [3], and the bacterium has since adapted to land mammals, and the seal strains are surprisingly similar, despite species and geographical differences. The third cluster is somewhat curious. The isolate from the otter might indicate a species jump from a seal to land-bound mammals at a previous timepoint, and specifically a mustelid, as they seem to be susceptible to the bacterium and will come into contact with the aquatic environment through hunting for food. The same strain being found in a farmed mink in Spain is less easily explained and requires further study.

### 3.3. Pan, Core and Accessory Genomes of 42 A. phocae Strains

**Figure 2:**
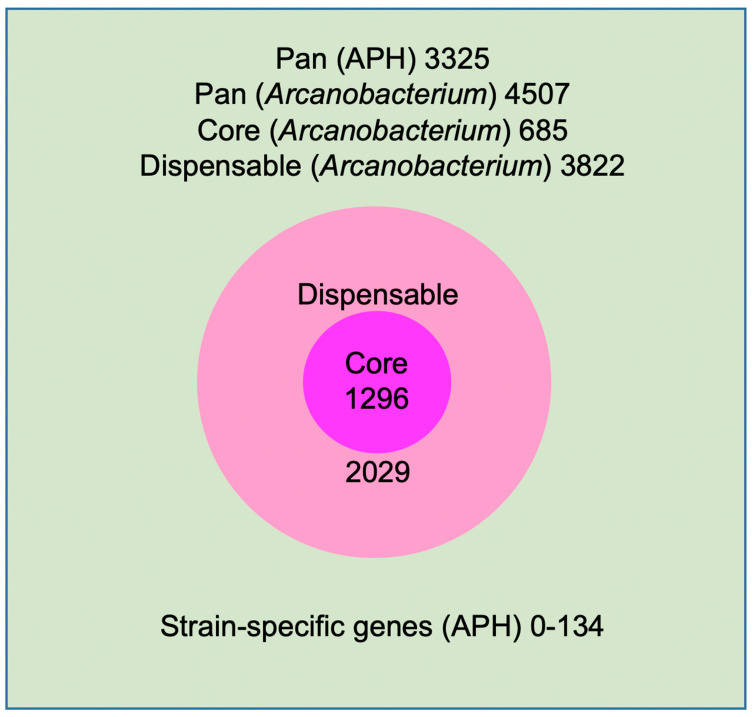
*A. phocae* pan-genome (3325 genes).

The genome sequences of 42 *A. phocae* strains were used to create the pan genome. The various genetic loci from the pan genome, essential for the survival of bacteria, forms the core genome of an individual species. These genes are necessary for the basic housekeeping of cellular functions, and also for metabolic, catabolic, transport activities, degradation of nucleic acids and ribosomes [24,25]. These 42 genomes formed an *Arcanobacterium phocae* pan genome of 3325 genes (Supplementary Table S1), of which only 39% (1296 genes) formed the core-genome (Supplementary Table S3), revealing a rather high inter-species diversity (Figure 2) [26,27,28,29]. We then calculated a similar pan- and core genome at the genus level with other closely related Arcanobacteria. The core dropped to 685 genes, but the pan genome went up to 4507 genes, highlighting the great variability and adaptability of this genus.

**Figure 3:**
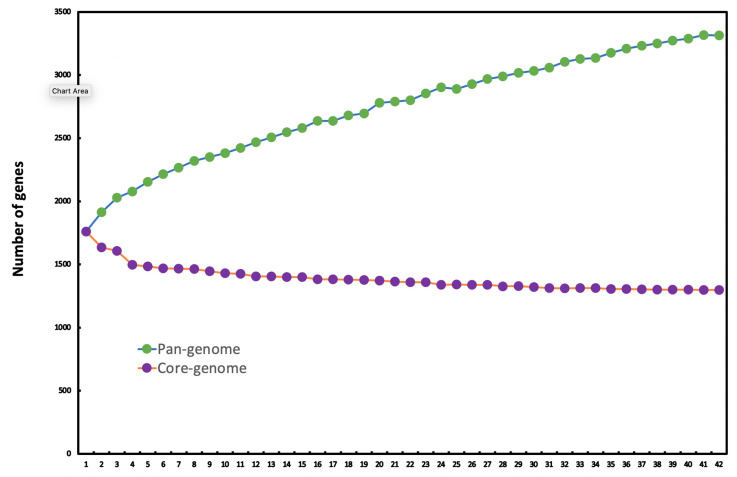
Pan-genome development plot of *A. phocae*.

When the number of genes in the *A. phocae* pan genome was plotted against the number of *A. phocae* genomes using the Heap’s Law calculation [24,25] the obtained α-value of 0.804 indicated that the pan-genome is still open but leveling out considerably (Figure 3). When examined closely, the pan genome development data curve seemed to be level at the start, at just over 3000 genes, implying that the pan genome of *A. phocae* is eventually proceeding towards a closed status. The addition of a few more strains would eventually close the pan genome representing the entire genetic repertoire of this population. It is vital to remember that we are only covering two known ecological niches with clear differences, and thus the core and pan genomes represent those strains, whilst others may exist. Similar trends were detected with the core genome development plot (Figure 3). The reasonably low number of core genes (1296) in this species suggests a wide-ranging genome structure, advocating a correspondingly large accessory genome. Even with the possible likelihood of a somewhat growing pan genome, *A. phocae* is an evolving species with multiple habitats. 

The part of the pan genome which is not included in the core is referred to as an accessory genome. These genes are non-essential but provide advantages to different strains and may hypothetically be part of the ecological adaptation. The accessory genome summaries the diversity of the species [24,25]. The 42 *A. phocae* strains form an accessory genome of 2029 genes (Figure 2), with 608 genes belonging to strain specific genes that can only be found in a single strain of *A. phocae* but are absent in all other strains (unique genes). Unique genes varied between 0 and 134 genes between 42 genomes. Remarkably, most of the dispensable genes were annotated as hypothetical proteins or proteins with an unknown function (Supplementary Table S1), and as most differences have unknown and uncharacterized functionalities, association with any type of adaptive role or benefits for the strains is difficult to show. However, the unique genes of a few strains were annotated with a range of predicted functions, from transport and metabolism to phage-related proteins and transposases.

### 3.4. Specific Genes

The 42 *A. phocae* strains contained 73 genes, which were absent in other genomes from this genus (Arcanobacterium). Most of these genes were hypothetical proteins, with few related to FMN reductase, coenzyme F420-dependent N5, N10-methylene tetrahydromethanopterin reductase, protein yceI precursor, ABC transporter ATP-binding protein, beta-galactosidase, endoglycoceramidase II, neuraminidase NanP, Na+/H+ antiporter, high-affinity choline uptake protein BetT, alkanal monooxygenase alpha chain, proline dehydrogenase, chloride channel protein, pirin, sialic acid transporter and error-prone repair homolog of DNA polymerase III alpha subunit proteins. In cluster one (including mink (*n* = 1) and otter (*n* = 1) isolate), 69 genes were specific to this clade, and most of them were found to be hypothetical proteins; therefore, the majority of the clearly annotated proteins were related to housekeeping and phage-associated proteins.

In clade two (including the five seal isolates), 12 genes were present, with eight hypothetical proteins, with one collagen-binding A protein (a virulence factor), one cytoplasmic protein and one arsenical resistance operon repressor. Cluster three consists of all isolates, except five isolates originating from the seal, otter or one mink (IN-5A). In cluster three, we found 27 genes with 17 hypothetical proteins, two membrane-related proteins, three related to mobile elements, two Tn5252-related proteins, one Sulfide:quinone oxidoreductase and one ATP-dependent DNA helicase RecG-related protein. These may be essential for niche adaptation and bacterial mutation for better receptor affinity, and the putative proteins, in particular, require further study.

### 3.5. Virulence Factors and Secreted Proteins for Putative Vaccine

Only 95 out of 1296 genes were found to be secreted in the core genome of the *A. phocae* strains. Sixty-one of these were SignalP-positive, while 34 were predicted to be lipoprotein-signal-peptide and three of the signal-peptide-positive genes also had the Tat signal (Supplementary Table S4). We also found a few *LPXTG*-like cell-wall proteins. The core also holds two toxins: phospholipase D and phocaelysin, which is a species-specific hemolysin with multiple homologs in other related bacteria, and was shown to be present in FENP-related *A. phocae* strains [30]. 

Virulence factors were analyzed separately from core- and pan-genomes. The virulence factors found in the pan genome are presented in Table 1. These factors were homologous to factors found in the genomes of mycobacteria, corynebacteria, streptococcae, listeria, and clostridia. When combined, the pan genome had 31 commonly recognized virulence factors but there could be more, as many proteins remain putative at this point.

Many virulence factors enable better adaptation to variable environmental stressors, but the most represented category is adherence-related genes: 17% of all virulence factors.

There are fewer virulence factors present in the core genome, with 23 factors. They are presented in Table 2. Most losses are seen in the adherence-enabling genes and surface-protein-anchoring genes, the most significant loss is of the two sortases. The pan genome also has genes for sortase-dependent pili, which are significant, among other functions, in adherence and, thus, virulence factors. This would also make them potential vaccine candidates. In addition, both pan and core genomes hold genes for Flp pilus assembly (TadC and TadD), making it plausible that the bacterium retained pilus-forming abilities. Only one virulence factor separates the seal strains and fur animal strains, despite significant differences at the genomic level. This single virulence factor is the collagen-binding protein, which is significant for adherence, and may possibly be more relevant to the bacteria in the marine environment. The other gene present in seal strains but not in others has a regulatory function in the arsenic resistance group of genes, which may be important when acting against environmental stressors. Our second adherence-related protein missing in the core genome is found in the third phylogenetic group, formed by one otter and one mink strain. Choline-binding protein is only present in this group, suggesting some role in ecological niche adaptation.

## 4. Conclusions

We have studied *A. phocae* strains from two separate ecological niches. We found the species to be highly variable, but the adaptation to new host species and environments has resulted in relatively stable strains, which apparently have low inter-niche variation. Due to the clear separation between seal and fur animal clades, the data suggest that the bacteria may have made a single species jump into mink, possibly during the Canadian outbreak of 1997, and then, after an adaptation period, from the mink to finnraccoon and bluefox with relatively little genomic level adaptation. On the other hand, Alssahem et al. have studied differences between fur animal isolates of *A. phocae* from a single farm, comparing them to isolates from another farm, and they found multiple signs of rapid adaptation at SNP level [30,31]. If the fur animal strains are studied further, it is possible that more adaptation will be seen to occur between different fur-animal-species-derived isolates. The seal cluster is surprisingly uniform considering the time lapse (1995–2015), different species, and geographical differences between isolates (Scotland, Denmark) (Figure 1). It is also clearly separate from the fur animal cluster. The third cluster is interesting and may represent a second introductory event closer to the present time, possibly from marine mammals to wild mustelids or from feed to farmed mink. Despite this, the data suggest that any major concerns regarding protein source hygiene can be laid to rest.

The relatively high number of virulence factors in the pan genome may help to explain the high pathogenicity of this bacterium. Many of these factors enable efficient intrusion into the host organism and heightened uptake of host-derived nutrients. We found several factors enabling cell entry, which correlates well with the observed poor response to antibiotic treatment, as the pathogen is able to hide within the cells of the host organism. Further enabling this treatment evasion is the collaboration of the exotoxins, which affect the walls of the blood vessels, collapsing them. This also leads to the necrosis that is typical of FENP. There also seems to be a healthy number of factors enabling efficient adaptation to environmental stressors, as well as a few connected to biofilm formation, offering an explanation for the difficulties in biosecurity and redeeming already infected farms.

The large number of putative proteins in the core and pan genomes yields possibilities for further study as more protein functions are identified. This is of special interest, as most of the proteins separating the two major clusters are putative, thus making them of special interest in adaptation to new hosts and environments. The solution to the effective treatment of the epidemic by a specific vaccine may also be found here.

## Figures and Tables

**Table 1:** The virulence factors found in the pan genome of *A. phocae*.

Function	Virulence Factor					
Aminoacid and purine metabolism	Glutamine synthesis	Purine synthesis				
Anaerobic respiration	Nitrate reductase	Nitrate/nitrite transporter				
Cell surface components	Lipoprotein	Methyltransferase				
Iron uptake	Iron-dependent regulator	Siderophore-dependent iron uptake system *	Heme ABC-transporter ATP-binding protein			
Lipid and fatty acid metabolism	Lipase	Phospholipase C				
Magnesium uptake	Magnesium transport					
Phagosome arresting	Nucleoside-diphosphate kinase	Tyrosine phosphatase				
Protease	Proteasome-associated proteins					
Regulation	WhiB3	CheA/CheY				
Secretion system	ESX-1 (T7SS)					
Stress adaptation	Catalase-peroxidase cat	superoxide dismutase				
Adherence	Collagen-binding protein	Siderophore-dependent iron uptake system *	Cell surface hydrophobicity proteins	Sortase A *	Choline-binding proteins	GroEL
Toxin	Phospholipase D	Beta-hemolysin/cytolysin	CAMP factor			
Intracellular survival	Oligopeptide-binding protein					
Surface protein anchoring	Lipoprotein diacylglyceryl transferase	Sortase A *	Sortase B			

* Sortase A and Siderophore-dependent iron uptake system are indicated in two categories as they have functions in both.

**Table 2:** Virulence factors in the core genome of *Arcanobacterium phocae*.

Function	Virulence Factor		
Aminoacid and purine metabolism	Glutamine synthesis	Purine synthesis	
Anaerobic respiration	Nitrate/nitrite transporter		
Cell surface components	Lipoprotein	Methyltransferase	
Iron uptake	Siderophore-dependent iron uptake system	Iron-dependent regulator	Heme ABC-transporter ATP-binding protein
Lipid and fatty acid metabolism	Lipase	Phospholipase C	
Magnesium uptake	Magnesium transport		
Phagosome arresting	Nucleoside-diphosphate kinase	Tyrosine phosphatase	
Regulation	CheA/CheY	WhiB	
Secretion system	ESX-1 (T7SS)		
Stress adaptation	Superoxide dismutase		
Adherence	Siderophore-dependent iron uptake system	GroEL	
Toxin	Phospholipase D	Beta-hemolysin/cytolysin	CAMP-factor
Intracellular survival	Oligopeptide-binding protein		
Surface protein anchoring	Lipoprotein diacylglyceryl transferase		

## Data Availability

All 41 genomes are publicly available at NCBI.

## Supplementary Materials

The following are available online at https://www.mdpi.com/article/10.3390/antibiotics10060740/s1. Figure S1: A comparison of the 41 genomes; Table S1: List of the annotated genes predicted for the 41 newly sequenced genomes; Table S2: A general overview of the 42 Arcanobacterium phocae isolates and genomes; Table S3: Core-genome; Table S4: The secreted proteins found in the core genome of the 42 A. phocae isolates.
