# Supplementary material for: Comparative Genomics of 42 Arcanobacterium phocae Strains
Source: Antibiotics (Basel). 2021 Jun 18;10(6):740. doi: 10.3390/antibiotics10060740 (PMC8235330; doi:10.3390/antibiotics10060740)
Supplement: Supplementary file 1 [file antibiotics-10-00740-s001.zip › SupplementaryTableS2.pdf]

S-Table S2 A general overview of the 42 *Arcanobacterium phocae* isolates and genomes

Strain	Host source	Isolation location	Geographic region	Year	Accession nro	Status	Coverage	Contigs	Size (Mbps)	G+C (%)	ORFs	Proteins	Ref.
APH37	mink	skin	Ostrobothnia	2009	SAMN14609993	draft	89x	106	2.00	50.1	1997	1904	This study
APH40	finnraccoon	skin	Northern Ostrobothnia	2009	SAMN14609994	draft	41x	107	1.98	50.0	1962	1856	This study
APH41	blue fox	eye discharge	Northern Ostrobothnia	2009	SAMN14609995	draft	61x	106	1.98	50.0	1961	1859	This study
APH67	mink	eyelid	Central Ostrobothnia	2010	SAMN14609996	draft	49x	106	1.98	50.0	1953	1853	This study
APH81	mink	skin	Central Ostrobothnia	2010	SAMN14609997	draft	56x	86	1.97	50.0	1947	1870	This study
APH96	finnraccoon	paw pad	Northern Ostrobothnia	2011	SAMN14609998	draft	66x	207	2.05	50.6	2091	2005	This study
APH98	blue fox	eye discharge	Pirkanmaa	2011	SAMN14609999	draft	70x	92	1.97	50.0	1961	1876	This study
APH105	mink	skin	Central Ostrobothnia	2012	SAMN14610000	draft	65x	99	1.98	50.0	1957	1865	This study
APH107	mink	paw pad	Central Ostrobothnia	2012	SAMN14610001	draft	55x	109	1.98	50.0	1977	1885	This study
APH122	mink	lung	Ostrobothnia	2013	SAMN14610002	draft	72x	93	1.97	50.0	1943	1853	This study
APH127	mink	lung	Ostrobothnia	2014	SAMN14610003	draft	91x	101	1.99	50.2	1989	1889	This study
APH130	mink	paw pad	Northern Ostrobothnia	2015	SAMN14610004	draft	86x	111	1.98	50.0	1971	1869	This study
DSM 10002	seal	lung	coastal waters (Scotland, UK)	1995	SAMN04489737	complete	882x	1	2.00	50.0	1801	1697	Unpublished
52753	finnraccoon	claw	Denmark	2013	SAMN14610006	Draft	66x	29	1.93	50	1740	1687	This study

12814	mink	fluid thorax	Denmark	2015	SAMN14610007	Draft	62x	40	1.92	50	1726	1673	This study
12817	mink	skin	Denmark	2015	SAMN14610008	Draft	60x	28	1.93	50	1742	1689	This study
12824	mink	digit	Denmark	2015	SAMN14610009	Draft	51x	40	1.93	50	1738	1684	This study
13645	mink	lungs	Denmark	2015	SAMN14610010	Draft	64x	23	1.94	50.1	1772	1718	This study
13808	mink	thorax	Denmark	2015	SAMN14610011	Draft	96x	26	1.93	50	1741	1688	This study
14235	mink	skin	Denmark	2015	SAMN14610012	Draft	76x	30	1.92	50	1736	1683	This study
20171	otter	abscess	Denmark	2015	SAMN14610013	Draft	92x	11	1.86	50.1	1636	1584	This study
22705	spotted seal	pharynx	Denmark	2015	SAMN14610014	Draft	68x	24	1.96	49.9	1733	1680	This study
22711	grey seal	claw	Denmark	2015	SAMN14610015	Draft	68x	14	1.87	50.1	1671	1618	This study
11692	mink	nose	Denmark	2017	SAMN14610016	Draft	97x	31	1.93	50	1743	1689	This study
22984	spotted seal	lesion (right back flipper)	Denmark	2015	SAMN14610017	Draft	57x	19	1.88	50.1	1675	1620	This study
22985	spotted seal	lesion	Denmark	2015	SAMN14610018	Draft	70x	143	2.04	49.9	1827	1777	This study
4114	mink	digit	Denmark	2015	SAMN14610019	Draft	78x	36	1.92	50	1733	1680	This study
4544	mink	skin	Denmark	2015	SAMN14610020	Draft	69x	23	1.99	50.4	1820	1766	This study
11893	mink	lung	Denmark	2017	SAMN14610021	Draft	103x	27	1.93	50	1737	1684	This study
12172	mink	fluid	Denmark	2017	SAMN14610022	Draft	70x	27	1.93	50	1746	1690	This study
14592	mink	lungs	Denmark	2017	SAMN14610023	Draft	57x	25	1.94	50.1	1769	1715	This study
ARC106	mink	lung	Finland	2012	SAMN14610024	Draft	73x	23	1.95	50.1	1770	1717	This study

ARC109	mink	skin	Finland	2013	SAMN 14610025	Draft	57x	29	1.93	50	1742	1687	This study
ARC127	mink	lung	Finland	2014	SAMN 14610026	Draft	74x	20	1.94	50.1	1767	1714	This study
ARC131	mink	absces s	Finland	2015	SAMN 14610027	Draft	68x	29	1.93	50	1738	1685	This study
SBAK872	mink	skin	Finland	2016	SAMN 14610028	Draft	74x	17	1.94	50.1	1760	1707	This study
ARCANO14	mink	UN	The Netherland	2012	SAMN 14610029	Draft	72x	31	1.94	49.9	1745	1692	This study
ARCANO19	mink	UN	The Netherland	2013	SAMN 14610030	Draft	70x	31	1.94	50	1752	1698	This study
ARCANO21	mink	UN	The Netherland	2013	SAMN 14610031	Draft	87x	20	1.95	50.1	1775	1722	This study
IN-1A	mink	UN	Spain	2014	SAMN 14610032	Draft	61x	21	1.95	50.1	1797	1746	This study
IN-4A	mink	UN	Spain	2015	SAMN 14610033	Draft	43x	19	1.94	50.1	1766	1714	This study
IN-5A	mink	UN	Spain	2015	SAMN 14610034	Draft	70x	13	1.86	50.1	1636	1581	This study
