# Supplementary material for: Comparative Genomics of 42 Arcanobacterium phocae Strains
Source: Antibiotics (Basel). 2021 Jun 18;10(6):740. doi: 10.3390/antibiotics10060740 (PMC8235330; doi:10.3390/antibiotics10060740)
Supplement: Supplementary file 1 [file antibiotics-10-00740-s001.zip › SupplementaryTableS4.pdf]

S-Table S4. The secreted proteins found in the core genome of the 42 *A. phocae* isolates.

BLT51_RS00050	SignalP-5.0	signal_peptide	.	phosphatidylserine/phosphatidylglycerophosphate/ cardiolipin synthase family protein
BLT51_RS00110	SignalP-5.0	signal_peptide	.	ABC transporter permease
BLT51_RS00165	SignalP-5.0	signal_peptide	.	hypothetical protein
BLT51_RS00260	SignalP-5.0	lipoprotein_signal_peptide	.	hypothetical protein
BLT51_RS00320	SignalP-5.0	lipoprotein_signal_peptide	.	maltose ABC transporter substrate-binding
BLT51_RS00340	SignalP-5.0	lipoprotein_signal_peptide	.	hypothetical protein
BLT51_RS00345	SignalP-5.0	signal_peptide	.	hypothetical protein
BLT51_RS00480	SignalP-5.0	signal_peptide	.	HlyC/CorC family transporter
BLT51_RS00505	SignalP-5.0	lipoprotein_signal_peptide	.	ABC transporter substrate-binding protein
BLT51_RS00520	SignalP-5.0	signal_peptide	.	hypothetical protein
BLT51_RS00615	SignalP-5.0	signal_peptide	.	hypothetical protein
BLT51_RS00715	SignalP-5.0	signal_peptide	.	NADH-quinone oxidoreductase subunit A
BLT51_RS00840	SignalP-5.0	signal_peptide	.	glycerol-3-phosphate dehydrogenase subunit GlpB
BLT51_RS00955	SignalP-5.0	signal_peptide	.	D-alanyl-D-alanine D-alanyl-D-alanine-endopeptidase
BLT51_RS00965	SignalP-5.0	signal_peptide	.	hypothetical protein
BLT51_RS00995	SignalP-5.0	signal_peptide	.	bifunctional metallophosphatase/5-nucleotidase
BLT51_RS01005	SignalP-5.0	signal_peptide	.	hypothetical protein
BLT51_RS01185	SignalP-5.0	lipoprotein_signal_peptide	.	hypothetical protein
BLT51_RS01210	SignalP-5.0	lipoprotein_signal_peptide	.	ABC transporter substrate-binding protein
BLT51_RS01215	SignalP-5.0	signal_peptide	.	hypothetical protein
BLT51_RS01315	SignalP-5.0	signal_peptide	.	hypothetical protein
BLT51_RS01355	SignalP-5.0	signal_peptide	.	tryptophan-rich sensory protein
BLT51_RS01370	SignalP-5.0	signal_peptide	.	hypothetical protein
BLT51_RS01425	SignalP-5.0	lipoprotein_signal_peptide	.	sugar ABC transporter substrate-binding protein
BLT51_RS01455	SignalP-5.0	lipoprotein_signal_peptide	.	thiamine ABC transporter substrate-binding protein
BLT51_RS01490	SignalP-5.0	lipoprotein_signal_peptide	.	zinc ABC transporter substrate-binding protein
BLT51_RS01500	SignalP-5.0	signal_peptide	.	type I 3-dehydroquinate dehydratase

BLT51_RS01640	SignalP-5.0	lipoprotein_signal_peptide	.	ABC transporter substrate-binding protein
BLT51_RS01870	SignalP-5.0	signal_peptide	.	DUF2142 domain-containing protein
BLT51_RS02030	SignalP-5.0	lipoprotein_signal_peptide	.	hypothetical protein
BLT51_RS02180	SignalP-5.0	signal_peptide	.	hypothetical protein
BLT51_RS02205	SignalP-5.0	signal_peptide	.	hypothetical protein
BLT51_RS02210	SignalP-5.0	signal_peptide	.	hypothetical protein
BLT51_RS02330	SignalP-5.0	signal_peptide	.	hypothetical protein
BLT51_RS02385	SignalP-5.0	signal_peptide	.	hypothetical protein
BLT51_RS02425	SignalP-5.0	signal_peptide	.	hypothetical protein
BLT51_RS02965	SignalP-5.0	signal_peptide	.	metallophosphoesterase
BLT51_RS02970	SignalP-5.0	signal_peptide	.	peptidoglycan glycosyltransferase
BLT51_RS03075	SignalP-5.0	lipoprotein_signal_peptide	.	alpha/beta hydrolase
BLT51_RS03240	SignalP-5.0	signal_peptide	.	hypothetical protein
BLT51_RS03255	SignalP-5.0	signal_peptide	.	TQXA domain-containing protein
BLT51_RS03290	SignalP-5.0	signal_peptide	Note=TAT	hypothetical protein
BLT51_RS03320	SignalP-5.0	signal_peptide	.	hypothetical protein
BLT51_RS03385	SignalP-5.0	signal_peptide	.	hypothetical protein
BLT51_RS03415	SignalP-5.0	signal_peptide	.	hypothetical protein
BLT51_RS03600	SignalP-5.0	signal_peptide	.	Na+/H+ antiporter NhaA
BLT51_RS03730	SignalP-5.0	lipoprotein_signal_peptide	.	DUF4300 domain-containing protein
BLT51_RS03805	SignalP-5.0	lipoprotein_signal_peptide	.	rhodanese-like domain-containing protein
BLT51_RS03835	SignalP-5.0	signal_peptide	.	HPr family phosphocarrier protein
BLT51_RS03840	SignalP-5.0	signal_peptide	.	phosphoenolpyruvate--protein phosphotransferase
BLT51_RS03875	SignalP-5.0	signal_peptide	.	L-lactate dehydrogenase
BLT51_RS03880	SignalP-5.0	lipoprotein_signal_peptide	.	LppP/LprE family lipoprotein
BLT51_RS03920	SignalP-5.0	signal_peptide	.	ABC transporter permease
BLT51_RS03935	SignalP-5.0	lipoprotein_signal_peptide	.	hypothetical protein
BLT51_RS04010	SignalP-5.0	signal_peptide	.	LytR family transcriptional regulator
BLT51_RS04030	SignalP-5.0	signal_peptide	.	hypothetical protein
BLT51_RS04055	SignalP-5.0	lipoprotein_signal_peptide	.	hypothetical protein
BLT51_RS04145	SignalP-5.0	signal_peptide	.	50S ribosomal protein L11

BLT51_RS04405	SignalP-5.0	signal_peptide	.	hypothetical protein
BLT51_RS04705	SignalP-5.0	lipoprotein_signal_peptide	.	hypothetical protein
BLT51_RS04785	SignalP-5.0	lipoprotein_signal_peptide	.	BMP family ABC transporter substrate-binding protein
BLT51_RS04950	SignalP-5.0	signal_peptide	.	hypothetical protein
BLT51_RS05040	SignalP-5.0	lipoprotein_signal_peptide	.	phosphate ABC transporter substrate-binding protein PstS
BLT51_RS05155	SignalP-5.0	signal_peptide	.	bifunctional metallophosphatase/5-nucleotidase
BLT51_RS05220	SignalP-5.0	lipoprotein_signal_peptide	.	hypothetical protein
BLT51_RS05250	SignalP-5.0	signal_peptide	.	endonuclease/exonuclease/phosphatase family protein
BLT51_RS05260	SignalP-5.0	lipoprotein_signal_peptide	.	metal ABC transporter substrate-binding protein
BLT51_RS05355	SignalP-5.0	signal_peptide	.	thiol:disulfide interchange protein
BLT51_RS05360	SignalP-5.0	lipoprotein_signal_peptide	.	hypothetical protein
BLT51_RS05665	SignalP-5.0	signal_peptide	.	phospholipase
BLT51_RS05765	SignalP-5.0	lipoprotein_signal_peptide	.	carbohydrate ABC transporter substrate-binding protein
BLT51_RS05850	SignalP-5.0	signal_peptide	.	hypothetical protein
BLT51_RS05855	SignalP-5.0	signal_peptide	.	HNH endonuclease
BLT51_RS05965	SignalP-5.0	signal_peptide	.	hypothetical protein
BLT51_RS06075	SignalP-5.0	signal_peptide	.	hypothetical protein
BLT51_RS06095	SignalP-5.0	lipoprotein_signal_peptide	.	ABC transporter
BLT51_RS06145	SignalP-5.0	signal_peptide	Note=TAT	DUF853 domain-containing protein
BLT51_RS06320	SignalP-5.0	lipoprotein_signal_peptide	.	ABC transporter substrate-binding protein
BLT51_RS06480	SignalP-5.0	signal_peptide	.	copper transporter
BLT51_RS06715	SignalP-5.0	lipoprotein_signal_peptide	.	hypothetical protein
BLT51_RS06940	SignalP-5.0	lipoprotein_signal_peptide	.	ABC transporter substrate-binding protein
BLT51_RS07050	SignalP-5.0	signal_peptide	.	MMPL family transporter
BLT51_RS07135	SignalP-5.0	signal_peptide	.	protein translocase subunit SecD
BLT51_RS07185	SignalP-5.0	lipoprotein_signal_peptide	.	phosphatidylinositol mannoside acyltransferase
BLT51_RS07445	SignalP-5.0	signal_peptide	.	hypothetical protein
BLT51_RS07485	SignalP-5.0	lipoprotein_signal_peptide	.	hypothetical protein
BLT51_RS07670	SignalP-5.0	signal_peptide	.	TIGR01777 family protein
BLT51_RS07735	SignalP-5.0	lipoprotein_signal_peptide	.	sugar-binding protein
BLT51_RS07990	SignalP-5.0	signal_peptide	Note=TAT	damage-inducible protein CinA

BLT51_RS08075	SignalP-5.0	lipoprotein_signal_peptide	.	iron ABC transporter substrate-binding protein
BLT51_RS08275	SignalP-5.0	signal_peptide	.	M23 family peptidase
BLT51_RS08465	SignalP-5.0	signal_peptide	.	hypothetical protein
BLT51_RS08695	SignalP-5.0	signal_peptide	.	hypothetical protein
BLT51_RS08715	SignalP-5.0	lipoprotein_signal_peptide	.	hypothetical protein
BLT51_RS08910	SignalP-5.0	lipoprotein_signal_peptide	.	hypothetical protein
