# Supplementary figures and images for: Comparative Genomics of 42 Arcanobacterium phocae Strains
Source: Antibiotics (Basel). 2021 Jun 18;10(6):740. doi: 10.3390/antibiotics10060740 (PMC8235330; doi:10.3390/antibiotics10060740)

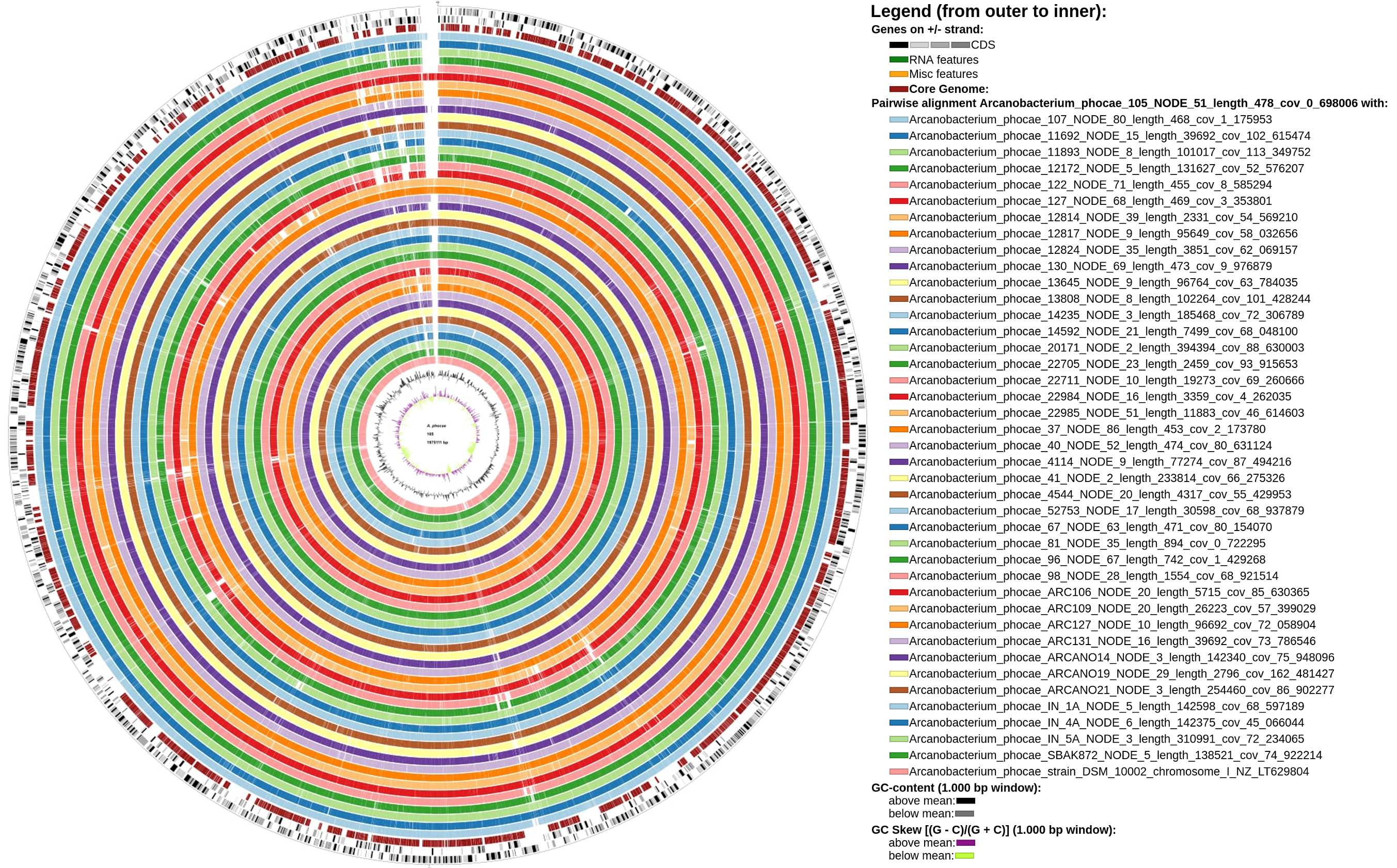

Supplement: Supplementary file 1 [file antibiotics-10-00740-s001.zip › SupplementaryFigureS1.png]
